# Noncompetitive Gametic Isolation between Sibling Species of Cricket: A Hypothesized Link between Within-Population Incompatibility and Reproductive Isolation between Species

**DOI:** 10.1155/2012/593438

**Published:** 2012-11-29

**Authors:** Jeremy L. Marshall, Nicholas DiRienzo

**Affiliations:** ^1^Department of Entomology, Kansas State University, 123 W. Waters Hall, Manhattan, KS 66506, USA; ^2^Department of Neurobiology, Physiology and Behavior, University of California, Davis, CA 95616, USA

## Abstract

Postmating, prezygotic phenotypes are a common mechanism of reproductive isolation. Here, we describe the dynamics of a noncompetitive gametic isolation phenotype (namely, the ability of a male to induce a female to lay eggs) in a group of recently diverged crickets that are primarily isolated from each other by this phenotype. We not only show that heterospecific males are less able to induce females to lay eggs but that there are male by female incompatibilities in this phenotype that occur within populations. We also identify a protein in the female reproductive tract that correlates with the number of eggs that she was induced to lay. Functional genetic tests using RNAi confirm that the function of this protein is linked to egg-laying induction. Moreover, the dysfunction of this protein appears to underlie both within-population incompatibilities and between-species divergence—thus suggesting a common genetic pathway underlies both. However, this is only correlative evidence and further research is needed to assess whether or not the same mutations in the same genes underlie variation at both levels.

## 1. Introduction

The link between intraspecific variation and interspecific divergence has been of general interest to evolutionary biologists since Darwin (e.g., [1–3]). More recently, researchers have been asking the question, does variation within the same genetic/physiological pathways, in the same genes, and, or at the same nucleotide position underlie both intraspecific and interspecific variation in a phenotype? These are important questions whose answers can provide insights into how reproductive isolation evolves.

For example, is the evolution of reproductive isolation so idiosyncratic that within-population variation and between-species divergence in the same phenotype are the by-product of different genes or pathways, thus yielding little predictability beyond the importance of the given phenotype? Or are there really genes, or pathways, that matter and are consistently involved in phenotypic variation at all levels (e.g., [4]). These two outcomes clearly represent the ends of a continuum and research is likely to find systems scattered across the whole spectrum. However, one of the goals of evolutionary biologists is to identify general patterns and, in this case, the goal should be to identify those kinds of phenotypes where a particular answer is likely.

To begin to add a data point to this discussion for a particular phenotype, we assessed the likelihood that a single genetic pathway may underlie a postmating, prezygotic phenotype that varies both within populations and between species. In particular, we examined a case of two cricket species (*Allonemobius socius* and *A. sp. nov.* Tex) that have diverged rapidly over the past 30,000 years [5–7] and are only isolated from one another by postmating, prezygotic phenotypes, including the reduced ability of heterospecific males to induce females to lay eggs [5]. Moreover, based on preliminary data and past experiments (e.g., [8, 9]), it appears that within a population there is a significant variation in the ability of individual males to induce females to lay eggs. 

Given these data, we did more extensive tests to determine the degree of within-population and between-species variation in this phenotype. Moreover, we identified and tested (with RNAi) a protein in the female reproductive tract that is correlated with this postmating, prezygotic phenotype. We found that the ability of a male to induce a female to lay eggs does vary within populations and between species, that a chemosensory protein in the female reproductive tract is directly correlated with this phenotype, and that failure to induce a female to lay eggs results in the same dysfunction of the female chemosensory protein whether the male is conpopulation or heterospecific. Overall, these data suggest that the same genetic and physiological pathways underlie both within-population incompatibilities and reproductive isolation between species. However, this is just a hypothesis that needs further testing to determine if the same mutations and genes influence both levels of variation.

## 2. Materials and Methods

### 2.1. A Noncompetitive Gametic Isolation Phenotype between Sibling Species

For the sibling species *A. socius* and *A. sp. nov.* Tex, previous research suggested that heterospecific males are less able to induce females to lay egg relative to their conspecific counterparts [5]. To further test this finding, we conducted crosses between a series of populations of each species. Specifically, we collected individuals from three populations of *A. socius* (AR30, collected in SW Arkansas; TX30/146, collected near Mt. Vernon, Texas; TX30/198, collected near New Boston, Texas) and three populations of *A. sp. nov.* Tex (TXIII2, near Taylor, Texas; TXIII3, near Hearne, Texas; TXIV6, near Lufkin, Texas). 

From these collections, juveniles were allozyme genotyped for species identification following Marshall [5] and reared to adulthood in sex-specific cages (following [10]). Virgin adults, 10–14 days posteclosion, were used in no-choice mating experiments following standard protocols for *Allonemobius* (see [9]). Females were either mated once with a conspecific or heterospecific male. In the case of the conspecific mating trials, the males were from a different population (i.e., heteropopulation). Following successful copulation, males were removed and females were allowed to oviposit for four days. The number of eggs laid by each female was counted after this four-day period. The resulting data for each cross-type were analyzed and the difference between con- and heterospecific egg laying for each population was assessed with a *t*-test.

In addition to the above crosses, we also conducted a set of con- and heterospecific crosses where we measured the length of time the male spermatophore (i.e., the spherical protein structure that contains the sperm and seminal fluid proteins that are passed to the female during copulation; see [11]) was attached to the female. Spermatophore attachment time in this case was estimated as copulation time plus the length of time the spermatophore was attached to the female following successful copulation [9]. These crosses used populations of *A. socius* from Georgia (GA985/22, near Cornelia, Georgia) and Missouri (Fenton, Missouri) and *A. sp. nov.* Tex from near Caddo Mills, Texas (TX30/87). 

These latter crosses were conducted and analyzed as above; however, spermatophore attachment time was used as a covariant. Additionally, following the egg-laying period, all females were frozen at −80°C and their reproductive tracts subsequently checked for the presence of the ejaculate. Also, all females were checked for sterility (i.e., no/few eggs present in the abdomen and no/few eggs laid) and the occurrence of egg reabsorption (a form of physiological senescence which is indicated by eggs turning brown within the female). If a female was sterile or reabsorbing her eggs then she was removed from the analysis. This approach yielded a scenario where successful copulations resulting in limited egg laying could be ascribed to an unsuccessful male-female interaction rather than sterility or reproductive senescence. The purpose of these crosses was to determine if there is a relationship between spermatophore attachment time and the number of eggs laid by a female. 

Lastly, we conducted a set of crosses using *A. sp. nov.* Tex (TX30/87) where we not only assessed the spermatophore attachment time but also the DNA concentration within the female reproductive tract following a single, successful copulation. The purpose of this experiment was to determine if there is a relationship between spermatophore attachment time and the amount of DNA in the female reproductive tract—a proxy for the amount of male ejaculate. If a positive relationship is found, then it can be assumed that longer attachment times result in a greater amount of ejaculate being transfered to the female—which may affect after copulation physiologies like egg laying.

### 2.2. Identification of a Protein Linked to Induction of Egg Laying

The interaction of the male ejaculate and female reproductive tract determines the success of a particular copulation and ultimately determines if after copulation physiologies are turned on. To begin to assess this male versus female dynamics, we did single, no-choice conspecific matings as outlined above using *A. socius* from GA985/22. After a successful copulation, females were given four days to lay eggs before being frozen at −80°C and their resulting laid eggs being counted. Next, the female reproductive tract (which includes the spermatheca and spermathecal duct) from each mated female was dissected out, placed in a 1.5 mL microcentrifuge tube with 20 *μ*L water, ground with a pestle and sonicated, and centrifuged at 15,000 rpms. The resulting supernatant from each male versus female interaction sample was assessed for protein concentration with a NanoDrop. Samples from each individual (50 *μ*g each) were run on a NuPAGE 4–12% Bis-Tris Gel with samples being arranged from the fewest eggs laid to the most eggs laid. After running and staining the gel, it was imaged and the relative abundance of particular proteins was assessed. Relative abundance was calculated by comparing the abundances of proteins of interest to a protein that was invariable across all samples. For each protein band on the gel, we determined whether or not the abundance of that protein correlated with the number of eggs laid by females. This protocol was repeated for heterospecific matings using male *A. sp. nov.* Tex from Terrell, Texas (TX20/RA).

From these gels, proteins of interest were identified with MS/MS following the protocols outlined in Marshall et al. [7, 11]. The resulting peptide data from MS/MS analyses were compared with our 454 EST library from the female reproductive tract of *A. socius*. For peptides that matched a sequence from our 454 library, we used BLASTp in NCBI to determine a possible identification of the proteins of interest. 

### 2.3. A Functional Genetic Test of a Female Reproductive Tract Protein

For the one protein whose abundance was correlated with patterns of egg laying, we conducted an RNAi experiment to test the function of this protein and determine if protein knockdown resulted in a phenotype consistent with the original correlative pattern. To accomplish this, we followed established RNAi protocols for *Allonemobius* [11]. In brief, we used female *A. socius* from GA985/22 and male *A. socius* and *A. sp. nov.* Tex from GA985/22 and TX20/RA, respectively. We generated dsRNA from a PCR template (~500 bp in length) using RNA polymerase and primers with a T7 promoter (T7 region is underlined; F primer, CSP1F, TAATACGACTCACTATAGGGAGAGAGCAGGTAGACACCTTCAT; R primer, CSP1R, TAATACGACTCACTATAGGGAGAGGAGGGTGTAAAAGGCTAAT). After cleaning the dsRNA product, we injected 1*μ*L of 1*μ*g/*μ*L dsRNA into the abdomen of a set of virgin females (that were <10 days posteclosion). As a control, we injected a separate set of virgin females with 1 *μ*L of saline. For the first six days postinjection we randomly sampled females from both treatments, dissected out their reproductive tracts, and ran the resulting protein samples on a protein gel to assess protein knockdown in the dsRNA treatment. Our focus was on the protein-level knockdown, rather than transcript-level knockdown, as it is the protein-level phenotype that was correlated with patterns of egg laying.

Following confirmation of successful protein-level knockdown, we conducted mating trials with the remaining females. Specifically, we conducted single, no-choice mating trials using both sets of females with some being mated to a conspecific male and others to a heterospecific male. Following successful copulation and spermatophore transfer, each female was given four days to lay eggs before being frozen and her eggs were counted. As above described, we determined the reproductive status of each female to remove the effects of sterility and senescence. The resulting egg-laying data were analyzed for the effects of RNAi knockdown.

## 3. Results

### 3.1. A Noncompetitive Gametic Isolation Phenotype between Sibling Species

**Figure 1:**
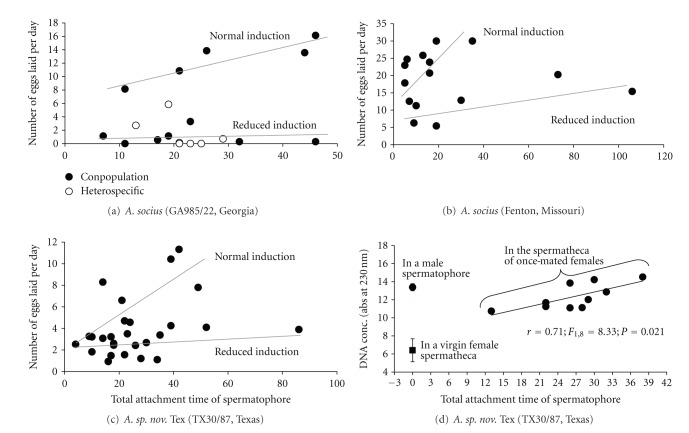
The relationship between spermatophore attachment time and egg laying for two populations of *A. socius* ((a) Georgia, USA; (b) Missouri, USA) and one population of *A. sp. nov.* Tex ((c) Texas). For each panel two patterns of egg laying are shown—the “normal induction” and “reduced induction” lines. Panel (d) shows the amount of ejaculate (measured by DNA concentration) that is passed to females during copulation. There are also two baseline measurements including the amount of DNA in a virgin female reproductive tract and in a male spermatophore.

For both species, successful heterospecific copulations (*N* = 150) result in females laying significantly fewer eggs compared with conspecific (but heteropopulation; *N* = 104) copulations (Table 1). Heterospecific copulations, relative to conspecific copulations, result in an average reduction of 75% to 99% in the number of eggs laid by a female *A. socius* and a 51% to 59% average reduction for females of *A. sp. nov.* Tex (Table 1). This pattern of heterospecific males being less able to induce a female to lay eggs is also seen when we control for the length of time the spermatophore is attached to the female (Figure 1(a)). In general, conspecific and heterospecific matings appear to have similar spermatophore attachment times—a finding also found for *A. socius* and *A. fasciatus* [9]. 

Interestingly, the standard deviation for all cross-types was large, indicating that individual crosses could result in no to hundreds of eggs being laid (Table 1). Part of this variation can be explained by the length of time the spermatophore was attached to the female (Figure 1(a), conpopulation cross). The “normal induction” line (a term used to signify that females were induced to lay eggs) shows a significant relationship between the total attachment time of the spermatophore and the number of eggs laid per day by a female for a conpopulation cross (*r* = 0.89; *F*_1,3_ = 11.53; *P* = 0.0426; Figure 1(a)). This relationship is consistent for distant populations of *A. socius* (Figures 1(a) and 1(b); populations from Georgia and Missouri) and for both species (Figures 1(a), 1(b), and 1(c)) and is likely driven by longer spermatophore attachment times resulting in a greater ejaculate transfer (Figure 1(d)).

However, the length of time the spermatophore is attached to the female cannot explain the large variance seen within conpopulation and conspecific crosses (Table 1 and Figures 1(a), 1(b), and 1(c)). Indeed, the “reduced induction” line (a term used to specify a cross where a male does not appear to have induced a female to lay her normal complement of eggs despite the successful transfer of ejaculate) shows that even if the spermatophore is attached for relatively long periods of time, a female may not be induced to lay eggs (Figures 1(a), 1(b), and 1(c)). This within-species pattern occurs in both species and suggests that a male versus female interaction underlies normal- or reduced-induction of egg laying. Moreover, at the phenotypic level, patterns of egg laying from heterospecific copulations resemble those of unsuccessful (i.e., reduced induction) conpopulation copulations (Figure 1(a)).

### 3.2. Identification of a Protein Linked to Induction of Egg Laying

**Figure 2:**
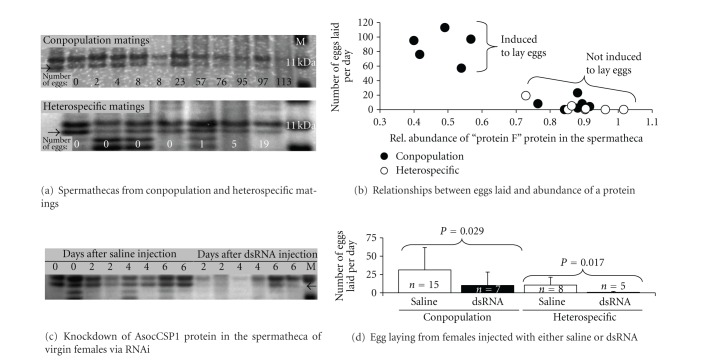
Patterns of variation and an RNAi experiment on protein F. (a) The abundance of protein F in the female reproductive tract following either a conpopulation (top) or heterospecific (bottom) copulation. (b) The measured relative abundance of protein F relative to patterns of egg laying for conpopulation and heterospecific copulations. (c) A gel showing that RNAi can knock down the abundance of AsocCSP1 (a.k.a. protein F). (d) An RNAi experiment showing patterns of egg laying in conspecific and heterospecific matings.

When running protein samples from the female reproductive tracts of mated females in the order of the fewest to the most eggs laid, we found that one protein (for the now called protein F) appeared to correlate with this pattern in both conspecific and heterospecific matings (Figure 2(a); see the arrow; *N* = 11). If we look at the relative abundance of protein F and patterns of egg laying, we find that when protein F is at reduced levels, females are induced to lay eggs, while the reverse is the case when protein F is at high levels (Figure 2(b)—data from Figure 2(a)). This pattern suggests that a successful copulation (i.e., one that will ultimately lead to egg laying) triggers protein F to be degraded or cross-linked or leave the female reproductive tract. Interestingly, heterospecific copulations yield patterns of protein F abundance that resemble those of conpopulation copulations where the female was not induced to lay eggs (Figure 2(b); *N* = 7). These data allow us to hypothesize that the same genetic and/or physiological pathway underlies both within-population incompatibility and reproductive isolation between species.

So, what is protein F? Using MS/MS, we identified two peptides of protein F that matched an EST in our female reproductive tract library (Table 2). We sequenced the underlying gene from cDNA derived from the female reproductive tract (NCBI accession number KC020194) and blasted the resulting sequence in NCBI and found that it matched a chemosensory protein in other insects (Table 2). Chemosensory proteins (abbreviated as CSPs) are small (~15 kDa) proteins that can reversibly bind small molecules/ligands and can be used to transport molecules from cell to cell or tissue to tissue within the body of insects [12]. Given these data, we propose to rename protein F as AsocCSP1. Interestingly, identifying this protein as a CSP suggests that the reduced abundance of this protein in the female reproductive tract following the successful induction of egg laying is likely the result of this protein leaving the female tract (and carrying the egg-laying stimulus) as opposed to being degraded or cross-linked.

### 3.3. A Functional Genetic Test of a Female Reproductive Tract Protein

To functionally test the correlation between AsocCSP1 abundance and egg laying in females, we conducted an RNAi experiment. Specifically, we wanted to test the prediction that if you knock down the abundance of AsocCSP1, that is, the protein that is hypothesized to carry the egg-laying stimulus out of the female reproductive tract, females would lay fewer eggs. To test this predication, we needed to knock down the abundance of the AsocCSP1 protein in the female reproductive tract. Using RNAi, we were able to successfully knock down the abundance of AsocCSP1 in the female reproductive tract of virgin females (Figure 2(c)). Given this success, we then mated both saline- and dsRNA-injected females with either a conpopulation or heterospecific male. We found that females injected with dsRNA laid significantly fewer eggs than females injected with saline when mated to a conspecific male (*P* = 0.029, Figure 2(d)). This pattern was also found when injected females were mated to heterospecific males (*P* = 0.017, Figure 2(d)). Additionally, we recovered the pattern that heterospecific males are less able to induce females to lay eggs (Figure 2(d)). Overall, this RNAi experiment supports the hypothesis that the AsocCSP1 protein is needed to successfully transmit the egg-laying induction signal from the female reproductive tract to a target elsewhere in the female's body. 

## 4. Discussion

Our mating experiments showed that heterospecific males of both species of *Allonemobius* are less able to induce females to lay eggs and that this reduced ability is likely the by-product of a male versus female incompatibility rather than incomplete ejaculate transfer (i.e., short spermatophore attachment times). These data are consistent with previous findings between *A. socius* and *A. fasciatus* [8], suggesting that this noncompetitive gametic isolation phenotype is common in this species complex. The finding that this form of postmating, prezygotic isolation is common in this species complex is not trivial. Indeed, besides conspecific sperm precedence, this mechanism of reproductive isolation is the only barrier to gene flow that is present in both the *A. socius-A. fasciatus* and *A. socius-A. sp. nov. *Tex contact zones. Therefore, given that this entire complex of crickets likely diverged over the past 30,000 years [5, 6], we have the opportunity to understand the evolution and genetic basis of reproductive isolation by studying this phenotype in this cricket complex.

Additionally, one of the most common postmating, prezygotic phenotypes is the ability of a male to induce a female to lay eggs (with over 29,000 publications indexed for “egg laying or oviposition” in Web of Science). Examples of this phenotype acting as a mechanism to reproductively isolate species can be found across the phylogeny of insects (e.g., green lacewings [13, 14], crickets [8, 15], walking sticks [16], beetles [17–19], wasps [20], and flies [21–23]). Therefore, our understanding of this phenotype in *Allonemobius* could shed light on a common mechanism of reproductive isolation in insects.

The importance of this postmating, prezygotic phenotype as a mechanism of reproductive isolation between species has overshadowed the occurrence of this phenotype within populations. For decades, mating experiments with these species have overlooked the fact that some matings just do not result in egg laying—writing it off as just something that happens (e.g., [24]). However, based on the more detailed analyses conducted here, it is clear that this pattern is the result of an incompatible male versus female interaction between the male ejaculate and female reproductive tract. This result is intriguing as it provides a hypothetical link between male-female incompatibilities within populations to divergence between species. In all, it was this hypothesis that prompted our search for a potential common genetic pathway. 

While the same phenotype was found both within populations and between species, it is important to remember that “similar phenotypes that vary within and between species may or may not be caused by the same genetic mechanisms” [4]. In this case, our findings are consistent with a common genetic/physiological pathway underlying this phenotype at both levels. It is important to remember, though, that this is only a hypothesis as we have yet to identify the exact mutations that result in this variation. However, our working hypothesis on how this genetic pathway functions is a two-step process. Step one, is a master on-off switch that results in the female being induced to lay eggs or not. More than likely, this is a male versus female allelic interaction between genes whereby the correct interaction turns on the after copulation egg-laying switch, while a dysfunctional interaction leaves the switch in the off position or only partially turned on—as if on a dimmer switch. Such a mechanism would explain variation at both the within-population and between-species levels. However, once the egg-laying switch is flipped on, then the amount of ejaculate (or a specific ejaculate substance) influences the number of eggs a female lays (as seen in Figure 1). Therefore, this second step is a dose-dependent step in which longer spermatophore attachment times, and thus greater ejaculate transfer, can result in more eggs being laid by the female. 

While our work provides justification for further testing this hypothesis, we still do not know if it is variation in the same genes (i.e., the actual male and female genes that interact) or mutations at the same nucleotide positions in those genes. Our next step is to identify the male and female genes that interact to initiate egg laying in these species and determine if mutations in the same genes and nucleotide positions account for our observed patterns. While the answers are still a few years out, our research points to a few G-protein coupled receptors in the female reproductive tract that interact with a set of peptides in the male ejaculate to initiate egg laying.

## Figures and Tables

**Table 1:** Con- and heterospecific mating crosses.

			Male population								
Comparison	Female species	Female population	Conspecific mating			Heterospecific mating			Statistics		
			Male	*N*	Number of eggs laid (SD)	Male	*N*	Number of eggs laid (SD)	Percentage of reduction	*t*-test	*P*_one tailed_
1	*A. socius *	AR30	TX198	14	116.1 (94.0)	TXIV6	19	1.4 (3.6)	98.8	5.19	<0.0001
2	*A. socius *	TX30/146	SC95/172	20	143.6 (61.6)	TXIII2	22	35.7 (53.1)	75.1	6.11	<0.0001
3	*A. socius *	TX198	TX30/146	9	70.6 (41.1)	TXIII2	28	13.5 (19.2)	80.9	5.74	<0.0001
4	*A. sp. nov.* Tex	TXIII2	TXIV6	22	142.4 (63.1)	TX198	48	58.5 (41.0)	58.9	6.66	<0.0001
5	*A. sp. nov. *Tex	TXIII3	TXIII2	25	89.5 (68.2)	TX30/146	17	43.4 (43.2)	51.5	2.47	0.0091
6	*A. sp. nov. *Tex	TXIV6	TXIII3	14	81.4 (74.8)	AR30	16	39.4 (38.0)	51.6	1.97	0.0293

**Table 2:** The identification of protein F with MS/MS and BLAST.

MS/MS Analyses			BLASTp info				
Peptides matched	Delta mass (Da)	MS/MS Ion score (*P* < 0.05*)	Hit number	Description	Organism	Accession number	*E* value
YDPQNLYAQAHPELFQ	−0.23	52*	1	Insect pheromone-binding family	*An* *op* *he* *le* *s*	XP_317405	1*E* − 11
QPQWEQIQK	−1.23	28			*ga* *mb* *ia* *e*		
			2	Chemosensory protein precursor	*Lo* *cu* *st* *a*	AAO16790	9*E* − 11
					*mi* *gr* *at* *or* *ia*		
